# Reply to “Reflections on the Hungarian oxford cognitive screen in post-stroke cognitive care”

**DOI:** 10.1016/j.cccb.2026.100557

**Published:** 2026-08-21

**Authors:** Tímea Tünde Takács, Panna Pálinkás, Bence Gunda

**Affiliations:** a Semmelweis University, Department of Neurology, Budapest, Hungary; b Semmelweis University, Faculty of Medicine, Budapest, Hungary

**Keywords:** Post-stroke cognitive impairment, Cognitive screening, Oxford cognitive screen, Stroke rehabilitation, Neuropsychological assessment, Stroke pathway

## Abstract

• Validation of the Hungarian Oxford Cognitive Screen has been completed, with publication forthcoming.
• Routine cognitive screening should be integrated into stroke care pathways where resources permit.
• Standardized screening may reveal unmet rehabilitation needs and inform healthcare policy.
• Comprehensive screening reduces the risk of missed post-stroke cognitive impairment.

We sincerely thank Dr. Polona Rus Prelog and colleagues [1] for their correspondence regarding our article detailing the Hungarian adaptation of the Oxford Cognitive Screen and its pilot study. We appreciate the authors' insights, which strongly reinforce the vision that has guided this project from its inception and closely align with our long-term objectives. In their commentary, the authors raise three main points, which we address below.

First, regarding the observation that linguistic adaptation must be followed by formal norming and cut-off derivation in the target populations, we are in complete agreement. We fully recognize that linguistic equivalence does not automatically guarantee psychometric equivalence. The primary objective of our pilot study [2] was to establish feasibility and cultural acceptability. We are pleased to report that the normative data collection has since been completed, and we are soon publishing a validation study, which will contain the cut-off scores and normative data for the Hungarian OCS.

Second, the authors highlight the importance of integrating screening into structured care pathways. We agree that screening is only a starting point and its true clinical value lies in leading to a comprehensive cognitive care pathway that includes targeted rehabilitation for domain-specific deficits [3]. However, one of the major challenges currently facing Hungarian stroke care is the extremely limited availability of outpatient neuropsychological training and cognitive rehabilitation services. As noted in domestic literature, long-term outcomes in Hungary are often affected by difficulties in post-discharge coordination, a lack of consistent early mobilization, and the growing shortage and high workload of healthcare professionals [4]. We believe that systematic implementation of standardized cognitive screening will not only improve individual patient care but also generate objective data on the true burden of post-stroke cognitive impairment. Demonstrating this unmet clinical need may provide important evidence for healthcare policymakers to support the expansion of neuropsychological services and cognitive rehabilitation programs within the national healthcare system.

Our next objective is to establish the Hungarian OCS as the standard cognitive screening instrument for stroke patients at Semmelweis University by implementing routine cognitive screening both during the acute phase of stroke and at the 3-month follow-up. We hope that demonstrating the feasibility and clinical value of this structured approach will promote its adoption as good clinical practice across Hungary.

Finally, we appreciate the suggestion that future studies should identify those patient populations deriving the greatest benefit from stroke-specific cognitive screening. While we agree that evaluating performance in patients with aphasia, neglect, severe strokes, and diverse lesion patterns is of considerable scientific and clinical importance, we would also advocate for the broadest possible implementation of cognitive screening whenever resources permit. Stroke-related cognitive impairment is highly heterogeneous, and neither lesion characteristics nor routine clinical variables reliably predict which individual patients will demonstrate clinically relevant cognitive deficits. Restricting screening to predefined subgroups therefore carries the risk of overlooking impairments in patients who might otherwise benefit from timely neuropsychological evaluation and rehabilitation. For this reason, our preferred strategy remains comprehensive screening of all stroke patients whenever feasible, while acknowledging that local staffing and organizational constraints may necessitate a more selective approach in some centres.

In summary, we thank Dr. Rus Prelog and colleagues for their valuable insights. Our ongoing work directly addresses the key points raised, including completed normative data collection and an upcoming full validation study. We aim to support the integration of the Hungarian OCS into routine stroke care and contribute to a more comprehensive post-stroke cognitive management system in Hungary.

## CRediT authorship contribution statement

**Tímea Tünde Takács:** Conceptualization, Writing – original draft, Writing – review & editing. **Panna Pálinkás:** Writing – original draft. **Bence Gunda:** Supervision, Writing – review & editing.

## Declaration of competing interest

The authors have nothing to declare.

## References

[1] Rus Prelog P., Zupan M., Frol S. (2026). Reflections on the Hungarian Oxford Cognitive Screen in post-stroke cognitive care. Cereb. Circ. Cogn. Behav..

[2] Takacs T.T., Karpati J., Szabo E., Palvolgyi K., Palinkas P., Antal O. (2026). The need for post-stroke cognitive screening - the rationale behind the Hungarian adaptation of the oxford cognitive screen (OCS) and its pilot study. Cereb. Circ. Cogn. Behav.

[3] Demeyere N. (2024). Acute post-stroke screening for a cognitive care pathway. Lancet Healthy Longev.

[4] Szocs I., Bereczki D., Belicza E. (2016). [Results of stroke care in Hungary in the frame of international comparison]. Orv. Hetil.

